# Polyphenolic Antibacterials for Food Preservation: Review, Challenges, and Current Applications

**DOI:** 10.3390/foods10102469

**Published:** 2021-10-15

**Authors:** Peter Martinengo, Kannappan Arunachalam, Chunlei Shi

**Affiliations:** Department of Food Science and Technology, School of Agriculture and Biology, and State Key Lab of Microbial Metabolism, Shanghai Jiao Tong University, Shanghai 200240, China; petermar@sjtu.edu.cn (P.M.); kannanacmdu@gmail.com (K.A.)

**Keywords:** plant polyphenols, antibacterial, foodborne pathogens, food preservation

## Abstract

Natural alternatives replacing artificial additives have gained much attention in the consumer’s view because of the growing search for clean label products that are devoid of carcinogenic and toxic effects. Plant polyphenols are considered as suitable alternative natural preservatives with antioxidant and antimicrobial properties. However, their uses in the food industry are undermined by a series of limitations such as low solubility and stability during food processing and storage, lack of standardization, and undesirable organoleptic properties. Different approaches in the use of polyphenols have been proposed in order to overcome the current hurdles related to food preservation. This review article specifically focuses on the antibacterial activity of plant-derived polyphenols as well as their applications as food preservatives, main challenges, and other trends in the food industry.

## 1. Introduction

Generally, the use of synthesized or unfamiliar substances in a food product leads the consumers to perceive the product as non-beneficial or toxic for their body and health. Therefore, several food additives such as genetically modified ingredients, artificial sweeteners, flavor-enhancers, food colorants, and preservatives contribute to create a negative image about the product [1]. Hence, this trend turns out the food industries to consider natural alternatives over the above-mentioned synthetic substances [2].

Several synthetic preservatives have been used to extend the shelf-life of the food products by inhibiting microbial growth. Moreover, specific food requires special attention against microbial spoilage along its preparation, storage and distribution, in order to avoid unpleasant effects (i.e., microbial growth, change in color, flavor, taste, texture) [3]. The presence of certain microorganisms (MO), such as *Salmonella* spp., *E. coli* O157:H7, *Listeria monocytogenes*, *Staphylococcus aureus*, *Campylobacter* spp., *Bacillus cereus*, and *Clostridium perfringens* questions the food quality, and also represents a potential hazard for causing foodborne diseases and their related outbreaks [4]. This is an increasing health issue worldwide. On a global scale, it is estimated that unsafe foods cause around 600 million of foodborne disease cases and 420,000 deaths each year [5].

When it comes to natural food preservatives, plants are regarded as the hub of bioactive secondary metabolites with such potentials. The major active compounds present in plants, herbs, and spices are phenolic compounds and their derivatives (flavonoids and non-flavonoids), terpenes, aldehydes, ketones, aliphatic alcohols, organic acids, thiosulfinates, saponins, and glucosinolates. Bioactive compounds present in plant extracts (PE) and essential oils (PEO) have the ability to delay the growth or inhibit the action of foodborne pathogens (bacteria, yeasts, molds, viruses) as shown in several recent studies [3,6,7,8]. Generally, the bioactive compounds from PEO and PE show better inhibitory activity on Gram-positive bacteria rather than Gram-negative bacteria. Apart from antibacterial activity, these bioactives might also exert antioxidant activity, rendering them valuable alternatives as commercial food preservatives [9]. As an alternative to synthetic preservatives, we have compiled broad information regarding polyphenolic classes and their fundamental characteristics in this review. Moreover, we also discussed the earlier reports on polyphenolic compounds showing antibacterial and antifungal activities, particularly against foodborne pathogens. Further, an overview of the industrial challenges in the application of plant polyphenols as food preservatives is provided with a special attention to the technologies and techniques, which can lessen the limitations.

## 2. Plant Polyphenols

Polyphenols represent one of the most widely distributed groups of compounds present in plants [10]. Polyphenols are considered as the non-nutritional secondary metabolites produced by plants through shikimate and/or polyketide pathways. The functions of polyphenols are largely varied across plants, such as pigment synthesis, protection against environmental stresses including UV radiations and pathogen attacks, and more often as chemical messengers [11]. The polyphenols are structurally abundant, and this structural diversity is well correlated for their distinctive properties such as bioactivity, stability, and bioavailability. Moreover, these compounds possess a better antioxidant and antimicrobial capacities, which may help to prevent foods from oxidative rancidity and microbial spoilage [2,12,13]. Further, the application of polyphenols as natural food preservatives is expected to rise above the ill effects of synthetic derivatives. Polyphenol-rich plants such as oregano, clove, green tea, citral, rosemary, thyme, grape, and sage have been tested for their antimicrobial activities against foodborne pathogens, either alone or in combination with other preservation techniques. 

## 3. Polyphenolic Antimicrobials

As a self-defence mechanism, plants produce secondary metabolites including polyphenols in response to microbial and other animals’ attack. Many studies showed the capability of polyphenols in inhibiting the growth of food spoilage bacteria, yeast, and fungi [2,12,14] (Table 1). The proven antimicrobial activity warrants the potential use of plant polyphenols as food preservatives [15]. Regarding the preservative potential of polyphenols in the food industry, several applications have been proposed. Moreover, each bioactive polyphenol has different action mechanisms, thus the bacteria are less likely to develop resistance against the polyphenols when used as combination with other antimicrobials and antibiotics. Thus, the use of polyphenols might reverse the trend and reduce the exposure of consumers to drug-resistant bacteria [16]. 

Polyphenolic compounds are the second largest family of plant-derived phytochemicals next to terpenes and terpenoids. Polyphenols share a typical chemical structure characterized by at least one aromatic ring with one or more hydroxyl groups as substituents [17]. To date, this family contains about 10,000 described compounds from vascular plants, hundreds of which are from herbal and edible plants, and spices [18]. This family can be sub-classified into smaller families depending on the chemical structure, the number of aromatic rings, and other structural elements such as the substituents linked to the rings. Common classifications are: (1) phenolic acids (i.e., gallic acid), (2) flavonoids (i.e., quercetin), (3) stilbenes (i.e., resveratrol), (4) lignans (i.e., secoisolariciresinol), (5) coumarins (i.e., coumarin), (6) tannins (i.e., proanthocyanidins), and (7) quinones (i.e., quinone) [19].

### 3.1. Flavonoids

Flavonoids are one of the sub-groups of polyphenols with low-molecular weight compounds. The compounds under the flavonoids class display wide range of pharmaceutical activities specifically antioxidant, antimicrobial activities and others. Flavonoids are present in the vascular strands of the plant, and have potential role in physiological regulations, and chemical signaling [78]. Among other functions, flavonoids are essential antimicrobials that have defensive activity in response to the pathogen assault [79]. Hence, the compounds under the group of flavonoids are termed phytoalexins and allelochemicals. As mentioned earlier, the core structure of flavonoids is composed of two benzene rings at C6(A)-C6(B) position bridged by oxygen-cyclized C3(C) carbon (C6-C3-C6, phenylbenzopyran) [80]. Based on the saturation and oxidation level, the flavonoids can be grouped into different subclasses such as flavones, isoflavones, flavonols, flavanols, flavanones, flavanonols, and chalcones [81]. It cannot be ignored that the constant alterations in the structure of flavonoids lead to numerous compounds with structural variety and broad biological activities [82]. The flavonoids glycosides and other derivatives of flavonoids including methoxylated, prenylated, and others vary in their structures and ultimately lead to the development of potential antimicrobial agents with novel targets [80] (Table 1).

#### 3.1.1. Flavones

The antibacterial properties of flavones are proposed with different action mechanisms. As one of the mechanisms, the flavones inhibit the adhesion and growth of cells through forming complex with the cell wall components. Following the similar mechanism, the flavones gancaonin Q [26] and amentoflavone (Mbaveng et al., 2008) inhibit the growth of *B. cereus* with MIC value of 2.4 and 39 µg mL^−1^, respectively. Similarly, the growth of *E. coli* was inhibited by licoflavone C through complex formation with extracellular and soluble proteins [27]. Apart from antibacterial activity, baicalein inhibit the virulence regulation of *S. aureus* by downregulating the quorum-sensing genes, *Staphylococcus* accessory regulator (*sarA*), and intercellular adhesins (*ica*) genes [21]. Potentiation of antibiotics is another mechanism, through which the flavones inhibit the bacterial growth. Through this mechanism, Artonin I targets the growth of *S. aureus* up to 69 to 89% [83]. 

#### 3.1.2. Flavonols

Flavonols consist of important class of antimicrobials including morin, quercetin, myricetin, rutin, kaempferol, and so on. Most of the compounds under the class flavonols are known for their antibacterial properties. Quercetin is a well-known polyphenol with GRAS status approved by the U.S. Food and Drug Administration [84]. A recent study by Wang and co-workers assessed the bacteriostatic effect of quercetin both in in vitro and in vivo conditions [39]. At in vitro condition, *E. coli*, *S. enterica* serotype Typhimurium, and *S. aureus* were assessed, and the minimum inhibitory concentration (MIC) of quercetin was found to be 0.0082, 0.0072, and 0.0068 µM mL^−1^, respectively. The pathogenic *E. coli* and *S. aureus* administrated with a higher MIC (50× and 10× MIC, respectively) of quercetin were found to disturb the cell wall and cell membrane of the respective pathogens, leading to the release of cytoplasmic content with reduced Adenosine 5’-triphosphate (ATP) production, synthesis of bacterial proteins, and finally to cell death. Morin is another flavonol present in plants such as *Psidium guajava*. The activity of morin is observed selectively against the Gram-positive and fungal pathogens. A study by Sivaranjani et al. demonstrated that morin have the potential to inhibit the biofilm formation of *L. monocytogenes* [36]. Further, the sub-MICs of morin reduced the pathogen load in the intestine of animal model, *Caenorhabditis elegans*, without showing any toxicity. A very recent work of Li et al. also demonstrated that morin targets the virulence regulation of the pathogen by inhibiting the major virulence factor listeriolysin O, which aids the cytosolic proliferation of bacteria inside the host by evading the host immune responses [85]. Besides the compounds enlisted, there are other compounds with potential antimicrobial activity against the foodborne pathogens, as listed in Table 1.

#### 3.1.3. Flavanones

3′,5-O-dimethyldiplacone, pinostrobin, sepicanin, 3′,5-di-O-methyl-diplacone, naringenin, mimulone, eriodictyol, pinocembrin diplacone, sophoraflavanones, pinocembrin 3′-O-methyldiplacol, and sakuranetin are some of the examples of compounds present in the flavanones subclass of flavonoids. The species from the *Dorstenia* genus was attempted to assess the antibacterial activity against *S. aureus* [29]. The flavanone compound 6,8-diprenyleriodictyol found responsible for the rapid bactericidal activity via depolarizing the cell membrane and inhibiting the biosynthesis of DNA, RNA and proteins. A lipophilic geranylated flavanone, mimulone, from *Heliotropium filifolium* was found active against both Gram-negative and Gram-positive bacteria [42]. The activity observed was superior when compared with that of positive control oxacillin. Synergism between antibiotics has been reported for the compounds under this subclass. For instance, sophoraflavanones (B and G) reported to have synergistic activity with conventional antibiotics such as ampicillin, oxacillin, and gentamycin against MRSA [86,87].

#### 3.1.4. Flavanols

Flavanols are often referred as flavan-3-ols, which contain skeleton with a 2-phenyl-3,4-dihydro-2*H*-chromen-3-ol. The main compounds under this class include mainly catechins, epicatechin, epigallocatechin, gallates of epicatechin and epigallocatechin, proanthocyanidins, theaflavins, and thearubigins. In general, catechins are frequently associated with antibacterial properties through their interactions with bacterial cell membrane. Unlike flavonoids, catechins have been found to tear bacterial membranes by attaching to the lipid bilayer and inactivating or inhibiting intracellular and extracellular enzymes [88]. According to Fathima and Rao (2016), it is also observed that catechins kill bacteria by producing reactive oxygen species (ROS) that change membrane permeability and destroy membranes [47]. Notably, oxidative bursts occur exclusively at high epigallocatechin, gallate concentrations. Grape seed extracts are rich in the monomers of catechins, epicatechin, and epicatechin gallates. A work by Levy and Co-workers demonstrated that grape seed extract inhibited the growth of foodborne pathogens *L. monocytogenes* and *S.* Typhimurium [89]. Because of the presence of negatively charged lipopolysaccharide on the outer membrane of the Gram-negative bacteria, catechins showed a high antibacterial tendency towards Gram-positive than Gram-negative bacteria [90,91].

#### 3.1.5. Chalcones

Several studies have delineated that compounds under the class of chalcones are associated with higher antibacterial efficacies or as potentiator of conventional antibiotics. 4-Hydroxyonchocarpin showed anti-staphylococcal activity with MIC value ranging from 1 to 8 µg mL^−1^ concentrations [29]. Similarly, isobavachalcone showed a very low MIC against *S. aureus*, which is a four-fold reduced concentration when comparing the conventional antibiotic gentamycin [20]. *Licorice* is a Chinese herb, which is known to produce more than 300 flavonoid molecules. Among these, the chalcones, licochalcone A and E, showed potent anti-staphylococcal (1–4 µg mL^−1^) and anti-candidal (0.2 µg mL^−1^) activities, respectively [51,52]. Further, these compounds reduced the biofilm formation and α-toxin production in *S. aureus*, and yeast to hyphal transition in *C. albicans*, respectively. 

### 3.2. Non-Flavonoids

The sub-group of non-flavonoids includes compounds with ample chemical structures, which vary from structurally simple molecules (i.e., phenolic acids and stilbenes) to highly complex molecules (i.e., stilbenes, lignans) [92]. Similar to the class of flavonoids, non-flavonoid compounds also display a wide range of pharmaceutical activities, including antimicrobial and antioxidant activities (Table 1).

#### 3.2.1. Phenolic Acids

In general, phenolic acids consist of phenolic compounds with one carboxylic acid substituent. Hydroxybenzoic acid and hydroxycinnamic acid are the two subgroups of phenolic acids [93]. Ferulic, caffeic, ρ-coumaric, vanillic, ρ-hydroxybenzoic, gallic, chlorogenic, and rosmarinic acids are some of the best studied phenolic acids. These phenolic acids have potent antibacterial activities and also being researched as food preservatives (Table 1). The presence of hydroxyl and methoxy group in the particular phenolic acid is responsible for the differences in their antibacterial activity. Further, the addition of alkyl chains is believed to increase the antimicrobial activity of the phenolic acids rather than their methyl and butyl esters [94,95]. The pH is an important factor in food preservation. The change in pH varies the antibacterial property of the phenolic acids by altering the charge of carboxyl group and substitution of hydroxyl and methoxy groups, and finally the side chain saturations [96]. A recent work by Pernin et al. [97] studied the antibacterial mechanism of eight phenolic acids such as chlorogenic acid, ferulic acid, gallic acid, ρ-hydroxybenzoic acid, protocatechuic acid, vanillic acid, caffeic acid, and ρ-coumaric acid against *L. monocytogenes* by quantifying the three different antibacterial factors such as decreasing the extracellular pH, and specific growth inhibition at undissociated or dissociated forms. The results of the work revealed that most of the polyphenols are active at their undissociated forms. The authors also suggest that antibacterial utility of the tested phenolic acids relies on their characteristics, especially pH. In another report of Gutiérrez-Larraínzar et al. [98], the authors assessed the antibacterial efficacy of p-hydroxybenzoic acid, protocatechuic acid, and gallic acid against *S. aureus, B. cereus*, *E. coli,* and *Pseudomonas fluorescens*. The results concluded that the Gram-positive organisms are more prone to phenolics than Gram-negative organisms. Gallic acid is the one with strong activity against *S. aureus*, and has a higher antioxidant potential tested with ferric reducing antioxidant power (FRAP), 2,2-diphenylpicrylhydrazyl (DPPH), and 2,2’-azino-bis(3-ethylbenzothiazoline-6-sulphonic acid (ABTS) assay than other phenolics tested [98].

#### 3.2.2. Stilbenes

Stilbenes consist of 1,2-diphenylethylene unit as their backbone. There are more than 400 stilbenes have been isolated and identified. Their therapeutic and preservative potentials are exemplified by compounds such as resveratrol and combretastatin [99]. Resveratrol (3,5,4′-trihydroxystilbene) is a naturally distributed phytoallexin, which is produced in plants such as grapes and grapevine berries in response to fungal infections by *Botrytis cinerea* and *Aspergillus carbonarius*, respectively [100,101]. Ma et al. analyzed the existing literature and reported that resveratrol showed inhibitory activity against wide range of foodborne pathogens including both Gram-positive and Gram-negative pathogens [62]. Combretastatins are class of compounds closely related to stilbenes. A derivative of combretastatin (B5) isolated from *Combretum woodii* leaf reported to have antistaphylococcal activity [60]. Similarly, Wu and Co-workers recently isolated a stilbene, longistylin A, from the leaves of *Cajanus cajan* [61]. The isolated stilbene showed rapid antibacterial activity, i.e., complete growth inhibition within 8 h of initial addition. The observed activity was found superior to the activity of last resort antibiotic vancomycin.

#### 3.2.3. Lignans

Lignans group of compounds are also known as phytoestrogens, which are produced from two phenylpropanoid units via oxidative dimerization process. This chemical family has two polypropanes (C6–C3) as their molecular backbone. These compounds are found in vascular plants belonging to more than 60 different families [102]. Different lignans such as justicidin B, vitrofolal C and D, (+)-epieudesmin, hinokinin, (+)-gmelinol, (+)-paulownin, (−)-taxiresinol, sesamin, and sesamol are reported to have significant antibacterial and antifungal activities [63,102]. Moreover, most lignans have strong antioxidant activity, which is still another important factor in food preservation [102].

#### 3.2.4. Coumarins

Coumarin is commonly called benzo-α-pyrone. This heterocyclic compound arose from the fusion of benzene nucleus with pyrone ring. Coumarins are classified based on the chemical compositions such as simple coumarins (umbelliferone), furanocoumarins (xanthotoxin and angeligin), pyranocoumarins (warfarin), and others [103]. Novobiocin from *Streptomyces niveus* and aflatoxin from *Aspergillus* sp. and chlorobiocin are well known examples of coumarins. There are many studies reporting the natural and synthetic derivatives of coumarins with antimicrobial activity, all of which have been meticulously reviewed by others [104].

#### 3.2.5. Tannins 

Tannins are one of the high molecular weight polyphenolic compounds, ranging between 500 to 30,000 Da. Tannins are mainly composed of two groups. Hydrolysable or pyrogallol tannins are mainly with phenolic acid esters and polyols. Gallotannins and ellagitannins come under hydrolysable tannins. The next group of tannins is condensed tannins or proanthocyanidins, and is resistant to acid hydrolysis. The simplest form of proanthocyanidins is procyanidins, which contains catechin or epicatechin [105]. In a study with hydrolysable tannins, Funatogawa and associates assess a total of 20 tannins and 6 proanthocyanidins against *Helicobacter pylori* and *E. coli*. All the hydrolysable tannins showed MIC values below 25 µg mL^−1^, except oenothein A, which displayed 5 µg mL^−1^ as its MIC. The derivatives of proanthocyanidins showed MIC at 100 µg mL^−1^ or more [68]. In addition to growth inhibition, tannins have been shown to inhibit the virulence by targeting biofilm formation, quorum sensing, virulence enzyme secretion, toxin production, and motility. Moreover, it is also reported that tannins potentiate the activity of antibiotics by inhibiting the activity of efflux pumps responsible for the development of multidrug-resistant strains [105].

#### 3.2.6. Quinones 

Quinone is a class of aromatic compounds. The structure of quinone is based on the number of benzene rings arranged in the skeleton. Basically, there are four types of quinones. They are benzoquinone (one benzene ring), napthoquinone (with two benzene rings), anthraquinone (with three rings in parallel), and phenantraquinone (three rings in different arrangement) [106]. This class of molecules is able to exert numerous biological functions, such as antimicrobial, antioxidant, antiviral, and antitumor effects. It has been reported that such activities of the compounds are related to the redox capacity of their carbonyl functions [107,108]. They can provide a source of free radicals and irreversible complexation with nucleophilic amino acids present in microbial proteins, resulting in the loss of the protein functions ultimately leading to cell death [109]. Thymoquinone, plumbagin, embelin, shikonin, salvicine, jugulone, and emodins are some examples of quinones with antibacterial efficacy [106].

Quinones, in particular naphthoquinones, are well known for their antibacterial and antifungal activities. This sub-class of polyphenols (i.e., plumbagin) was found efficient in targeting the functions of efflux pumps in Gram-negative bacteria [110]. Anthraquinones also have broad-spectrum antibacterial activity aiming the functions of bacterial proteins (i.e., adhesins), cell wall polypeptides, or enzymes which are positioned in/on the cell membrane of the target MO [111].

#### 3.2.7. Curcuminoid and Xanthanoids

Among polyphenols, curcumin is an extensively studied polyphenol with numerous biological activities, especially the ones that attract food industries such as the antimicrobial [74,112], antioxidant [113], and virulence inhibitory [114,115] properties. Curcumin is a hydrophobic, orange-yellow phenylpropanoids class of compound primarily extracted from *Curcuma longa*. It is a well-known drug in traditional Chinese and Indian medicine [116]. The antimicrobial efficacy of curcumin against foodborne pathogens has already been extensively investigated. The foodborne pathogens such as *B. cereus* and *B. subtilis*, *S. aureus*, *E. coli*, *L. monocytogenes*, and *Shigella dysenteriae* are few examples [112,117]. It has been also demonstrated that the other methods such as extraction, photodynamic inactivation, or exposure to UV strongly influence the antimicrobial activity of curcumin against *E. coli* and *Listeria innocua* and *L. monocytogenes*, and seems to have a potential application in the food industry [118,119,120]. Similarly, the derivatives of curcumin are also found to be effective against foodborne pathogens (Table 1).

Mangosteen (*Garcinia mangostana* Linn.) is a tropical tree from Southeast Asia. Mangostins belong to the class of isoprenylated xanthones (polyphenolic compounds), and are found to be abundant in the pericarp of the fruit. The chemical structure is composed of a tricyclic aromatic system having isoprene, hydroxyl, and methoxyl groups as substituents. Since the time of identification, several studies have been conducted in order to investigate the biological properties of these compounds, including antioxidant and other pharmacological activities [121,122]. Due to their hydrophobic characteristics, their bioavailability is quite limited, thus mangostins usually need a high content of surfactants [123]. Several pathogenic bacteria became very troublesome because of the emergence of antibiotic resistance. Staphylococci and Enterococci are one among the genera, which gained drug resistance during time. The antimicrobial action of α-mangostin has been tested against MRSA and vancomycin-resistant Enterococci (VRE) strains. The research of Koh et al. revealed the antibacterial action of α-mangostin against MRSA and *S. aureus*. The results showed MIC values varying from 0.78 to 1.56 µg mL^−1^ with rapid bacterial inhibition *in vitro* (3 log reduction in 5 min). The authors also claimed that the bioactive disrupts evolutionarily conserved bacterial membranes, resulting in its interesting antimicrobial effects [77]. These results suggested the efficacy of mangostin as an antibacterial agent and have the potential to get commercialized. Trying to improve the antibacterial efficacy and the selectivity of α-mangostin, the same research group in 2015 introduced different cationic amino acids and amine groups to the hydroxyl groups on C-3 and C-6 aiming to mimic the action of cationic antimicrobial peptides (CAMPs) [124]. According to this study, the newly formed structures carrying amino groups with high pKa values showed good antimicrobial activity and better selectivity. On the other hand, amino groups with low pKa values showed a reduced antimicrobial activity compared with α-mangostin. Likewise, Dharmaratne et al. conducted an assay on antimicrobial activity of γ-mangostin by testing its inhibitory effect against *S. aureus* and MRSA strains. The reported MIC values of γ-mangostin were 6.25 and 3.13 µg mL^−1^ for *S. aureus* and MRSA strains, respectively [125].

## 4. Technological Applications

### 4.1. Direct Incorporation

The direct incorporation of antimicrobial polyphenols into food showed to have significant growth inhibitory effects against pathogenic and spoilage MO [126,127,128]. The simplest incorporation method consists of mixing the natural antimicrobial with the other food ingredients. In case of a whole-piece solid food, the antimicrobial polyphenolic solution has to be placed onto the food surface either by dipping, spraying, or brushing methods [129]. The loss of efficacy due to the interaction with food components is the major problem encountered when directly incorporating the natural antimicrobial polyphenols into and onto the food system. Moreover, when the antimicrobial is mixed with other ingredients, it is difficult to achieve a homogeneous distribution, or to reach a sufficient amount of antimicrobial where the food system requires its action [126]. Davidson (1997) [130] described the following factors that affect the effectiveness of the incorporated antimicrobials in food systems. The first one is intrinsic factor, which is related to the characteristics of the food (i.e., pH, water activity, and composition). The second one is extrinsic factors, which is related to the environment (i.e., temperature, humidity, and atmosphere). The third one is with microbial factors, (i.e., initial number, lag time, growth rate, physiological status, drug resistance pattern, and the interactions between the MO) and finally the processing factors, which are related to the characteristics of food along the processing steps [130]. 

### 4.2. Encapsulation

Encapsulation is a process that is proposed to overcome the limitations such as bioavailability and stability of antimicrobial polyphenols in the food system [131]. In fact, this technique encapsulating the antimicrobials in a carrier molecule can increase their effectiveness by improving their distribution in the food matrix, which results in an increased product shelf life [132]. Encapsulation is extremely useful for the incorporation of hydrophobic compounds. Because of their nature, the distribution might not be homogeneous, or more concentrated in the lipid fraction. Instead, the microbial activity occurs in the hydrophilic portion, where the bioactive is required to prevent spoiling [133].

Among the encapsulation methods, spray drying, emulsification, solvent precipitation, electrospinning, and liposomes are the frequently used methods to form the micro or nanocapsules (Table 2). The encapsulation agent should be chosen carefully by considering the characteristics of the bioactive to be encapsulated. Moreover, it must be safe and authorized for human consumption. The most common natural materials used for the encapsulation of hydrophilic material are starch, alginate, glean, gelatin, carrageenan, xanthan gum, and milk proteins [134]. On the other hand, phospholipids are used to form liposomes or micelles encapsulating hydrophobic materials [133].

The encapsulation method is able to modulate the release of antimicrobial polyphenols into food in a controlled manner [146]. Several factors such as temperature, pH, water activity, food ingredients, type of preparation, and storage conditions can directly influence the release of the compound of interest [9]. The effectiveness of encapsulated antimicrobial compounds has been assessed against both the foodborne pathogens in food matrixes [146,147], and the shelf life of the commercial food products [148,149]. According to the research, the efficacy of this approach depends on the encapsulation process and materials employed, as well as the size of the encapsulated particle. Therefore, depending on the nature of the food, the particle size, coating material, and encapsulation method must all be carefully selected. Better results with higher antimicrobial efficacy were often obtained by using an encapsulating material with antimicrobial characteristics (i.e., chitosan) [150,151,152].

### 4.3. Edible Films and Coatings

This approach incorporates antimicrobial compounds into a layer of edible materials to distribute them directly onto the food surface. Edible films are preformed sheets, which are applied to the surface of the products, while edible coatings are formed by dipping, spraying, or spreading the food surface with coating solutions, allowing the formation of covering layers after the solutions have dried [153]. These approaches act as a protective cover against contaminations by pathogenic and spoilage bacteria. Moreover, these approaches can serve as a physical barrier between the food and the environment by preventing water loss and lowering the gaseous exchange. Moreover, the incorporation of bioactive compounds into films and coatings provides controlled release of the antimicrobial compounds and extends the shelf-life of the product [154] (Table 2).

The materials which serve as structural matrix for the preparation of edible films and coating may be obtained from different sources. They are mainly proteins, lipids, polysaccharides, or a combination of all of them [155]. Among the natural antimicrobials that can be incorporated in the structural matrixes, PEO and PE, bacteriocins, and enzymes have been recently studied [150,155,156]. In general, additional components such as plasticizers (i.e., glycerol, sorbitol) and cross-linking agents (i.e., transglutaminase) can also be added during the preparation. Other novel approaches such as nanoencapsulation and the development of a multilayered/nanolaminate delivery system might improve the antimicrobial effects of the materials incorporated in edible coatings [157].

The efficacy of edible coatings such as chitosan, hydroxypropylmethylcellulose, or sodium alginate, containing antimicrobial materials, was tested against perishable fresh foods with solid and semisolid characteristics (dairy products, wheat-based products, fish, meat, vegetables, and fruit). The incorporation of antimicrobials helped to control the development of pathogenic and spoilage MO on the product surface [151,152,155,156,157,158]. Among the above-mentioned matrix materials, chitosan proved to have optimal characteristics required for the preparation of edible coatings in food packaging [150,152,156].

This approach presents some challenges for its application in processed food, and a regulatory system must be developed in order to allow its use in the food industry [153]. An interesting and sustainable challenge would be to employ agroindustrial and food industry by-products as food matrix materials, so that they would not be considered industrial wastes [159]. A practical example was given by Palmeri el al., who employed prickly pear fruit extract both as structural matrix material and antimicrobial agent for the development of an edible coat in packed sliced meat [158].

### 4.4. Food Packaging

In active packaging, natural antimicrobials can be incorporated in different varieties of films, including traditional plastic based ones. This kind of packaging can also provide a controlled release of antimicrobials. Thus, this packaging combines the process of preservation and protection of food materials. Herein, the polyphenols can be immersed into the headspace of the package, or included into a sachet which allows the diffusion of antimicrobial polyphenols to the package headspace. In addition, certain antimicrobials may be used as non-release systems, and can be immobilized onto the inner surface of the packaging [129]. These approaches are gaining popularity in controlling microbial deterioration during storage [155] (Table 2).

This active packaging aims for the constant diffusion of the antimicrobials on the food surface, or its evaporation inside the headspace. Thus, determine the presence of the antimicrobials in a certain concentration for a long time during distribution and storage [157]. Concurrently, the release rate should be controlled in order to maintain the concentration and effectiveness of antimicrobials. This parameter also depends on the nature of the antimicrobial agent, packaging material, and food matrix [129]. Water-soluble and lipid-soluble antimicrobial agents are recommended in vacuum packaging, which contains high moisture and high lipid food, respectively. On the other hand, volatile antimicrobials have also been proposed for modified atmosphere packaged food with high surface-weight ratio [129,144].

## 5. Other Trends

Polyphenols might show synergism with antibiotics or other antimicrobials, which will improve the efficacy of antibiotics even at low dose [160,161,162]. For instance, natural polyphenols containing PE enhance the efficacy of antibiotics against multidrug-resistant pathogens such as MRSA and *K. pneumoniae* [163]. However, information about the interactions between specific combinations with different components in food is limited. Yet, the results obtained at lab conditions need validation in the food system the antimicrobial mechanism of the particular combination has to be further elucidated for their consistent use in food preservation. The successful results shown in vitro do not guarantee the same behavior in vivo [164]. Recently, a study aimed to investigate the antimicrobial activity of marjoram/lavender and marjoram/thyme essential oil combinations [165]. The results showed different levels of synergism. Further, the MIC value required to inactivate *S. aureus* and *E. coli* was significantly reduced when compared with the MIC of single essential oil. In particular for food items where the lavender, marjoram and thyme are typically used as flavoring agents, the use of such combinations may be regarded as natural alternatives to conventional food preservatives [165]. Similarly, an increase in the antimicrobial activity against *S.* aureus, *E. coli*, and *B.* cereus was observed for the combination kaempferol/resveratrol [166]]. Another option is to combine polyphenolic compounds with other kind of antimicrobials, such as nisin. A study conducted by Field et al., revealed the synergistic action of nisin V with carvacrol, thymol, and cinnamaldehyde against *L. monocytogenes* in a model food system [167]. Moreover, the combinations of nisin V with the antimicrobials showed better results against *L. monocytogenes* and *S. aureus* in the milk model. Yet, certain components present in the food matrix such as milk were reported to reduce the antimicrobial potential of the natural preservatives; the combination of nisin with natural preservatives showed promising activities [168]. Furthermore, Ayari et al. (2016) investigated the combination of carvacrol and nisin in controlling *B. cereus* in food following gamma-ray irradiation. After incorporating antimicrobials at a lower concentration into the food product, the use of prior technology resulted in a substantial reduction in pathogen load [169]. Synergism has also been shown between a bioengineered derivative on nisin (M21A), cinnamaldehyde, and citric acid in eradicating *L. monocytogenes* biofilms [170]. A recent trend revealed an increasing interest in the extraction of antimicrobials from woody parts of plants. For instance, antimicrobial and antibiofilm activities of laurel wood procyanidins were studied against foodborne bacteria. The combination of these polyphenols from laurel wood showed synergistic action against the growth and in biofilms of foodborne MO [171,172].

## 6. Challenges in the Application to Food Industry

Extraction of antimicrobial polyphenols from PE or PEO is the first hurdle to overcome. As polyphenols are concerned, the choice of the extraction method is crucial for maintaining their quantitative and qualitative properties. The efficiency of the extraction method depends on the solvent, the extractant, and the process [173]. For instance, the use of solvents such as methanol/water/HCL provided the best extraction of phenolic compounds from grape seeds [174], while acetone/acetic acid proved to be the better solvent to extract phenolics from basil leaves [175]. The implementation of additional procedures such as ultrahigh pressure [176], hydrodistillation [173] and ultrasound [174] might enhance the extraction efficacy of desired compounds. Since the use of natural food preservatives requires extraction and additional refining processes, they typically raise the manufacturing cost. Thus, the use of natural preservatives in food became more expensive compared with the artificial ones [177]. The use of polyphenol-rich PEO or PE as a food preservative also has certain limitations. Different quantitative and qualitative fluctuations in the content of PE and PEO question the reproducibility of their antimicrobial activity. Furthermore, mixtures are likely to contain toxic components. So, the adoption of polyphenol-rich PEO or PE for industrial use requires scrupulous investigation and standardization.

Next to the extraction methods, it is important to note the stability and bioavailability of polyphenolic compounds undergoing food processing procedures. It has been shown that temperature may affect the stability and consequently their activity during the storage time [178,179]. Likewise, interaction of polyphenols with the by-products produced during fermentation and drying processes can also reduce their bioavailability [180]. Hence, it is of prime importance to evaluate each and every step in food processing after adding polyphenolic antimicrobials to the food products.

When it comes to application, the effectiveness of antimicrobial polyphenols in food is the next prime challenge. Although polyphenols are reported to have better antimicrobial activity in lab conditions, their effectiveness in food systems may be negligible. Interaction of polyphenols with food macromolecules, such as carbohydrates, proteins, and lipids, is thought to be the cause of the observed detrimental effect [177,181]. Moreover, their use as food preservatives would require better toxicological information. Although certain polyphenols have been evaluated as “generally recognized as safe” (GRAS), the toxicological report about the use of such antimicrobials in food is not available. On the other hand, the addition of polyphenols might change the organoleptic characteristics of the food. Moreover, the concentration required for the antimicrobial packaging is much higher than the acceptable level, which may also raise the regulatory concern. To address this problem, the concentration of antimicrobial polyphenols needs to be dosed carefully in order to have the desirable effect without affecting the sensorial characteristics of the food [182].

The PE, PEO and individual polyphenols or in combination have several advantages in food packaging such as increased health, antioxidant, antimicrobial, UV barrier activities, and many more. In addition to the limitations discussed above, the uses of PE, PEO, and polyphenols have a few other limitations such as low solubility, high volatility, strong aroma, high sensitivity towards heat and light, and possibly have negative effects on the sensorial attributes of the food. Yet, the limitations in the use of polyphenols and polyphenol-rich PE and PEO in food packaging can be overcome by the utilization of different approaches, such as nanocarriers (nanoemulsions and nanoencapsulations) and different active packing methods. Further, the use of these approaches found much more effective along with the incorporation of additional technologies such as photodynamic inactivation, modified atmospheric packaging and others [183,184]. Many encapsulation matrices and techniques can be applied to fabricate natural preservatives into the nanocarriers. The fabrication of polyphenols or polyphenol rich PE and PEO itself is a challenging process, and depends on the nature of the bioactive, the presence of surface substituents, temperature, pH, degree of cellular uptake, and release [185]. In addition, certain low-energy fabrication methods require the utilization of materials such as solvents that might be toxic if not evaporated completely. Further, to obtain droplet size in nano-scale range for the efficient delivery of antimicrobial polyphenols in nanocarriers, it is necessitating the use the higher concentration of surfactant, and is a necessary in the process of nanoemulsion formation. Yet, the food packaging is highly regulated, and the food industries must oblige to follow the food packaging regulatory standards. Thus, it is very much critical to consider the properties of polyphenols to be fabricated, as well as to comply the regulatory daily intake of all compounds (i.e., polyphenols, surfactants, and others) used in the fabrication method [186,187].

Furthermore, antimicrobial agents migrating from food packaging materials to food are classified as food additives, and are required to meet the food additive standards. Besides high temperature and chemical affinity, the nature of food itself is a reason for the migration of natural antimicrobials. In order to assess the concentration of migration, migratory test may be performed to assess migratory potential of bioactive compound under given time and other storage conditions such as temperature and humidity. Additionally, while performing migration test, it is also important to consider the kind of polymer material and the migration compound. Food industries are obliged to follow the guidelines provided by regional competent authorities, such as the European Food Safety Authority (EFSA) in EU, the China Food Additives and Ingredients Association in China, and the Food and Drug Administration (FDA) in the US. Although there is no specific international regulation about the risk assessment of nano-products, regional authorities have established that food ingredients derived from nanotechnology application must be subject to a safety assessment prior to approval [188]. The ingestion of nano-scale additives might represent potential safety risks owed to their physiological and physicochemical mechanisms. It has been reported that the ingestion of metallic and inorganic nanoparticles might accumulate in various intestinal cells and tissues causing alterations in the functionality of the gastrointestinal tract. In contrast, organic nanoparticles are less likely to promote toxicity caused by absorption and accumulation in intestinal cells or other organs [189]. However, there are few works that studied the absorption, and the potential toxicity of organic nanoparticles. This is partially due to the difficulty in the detecting organic nanostructures within complex biological matrices containing similar components (such as polysaccharides, proteins, and lipids). For this purpose, future research might be focused on the development of suitable protocols and analytical methods. Since there are no clear regulations for nanofoods, a database for the released products was established in 2012. To date, the database contains information regarding 5224 products [190,191]

## 7. Conclusions

Because of the growing interest towards clean labeled products, the use of plant antimicrobials, especially polyphenols, as natural food preservatives is a promising trend in the industry. Several works currently assessed polyphenols in combination with other growth inhibitory compounds or technologies in order to extend the shelf life of products by controlling the growth of foodborne pathogens. On the other hand, the commercial adoption of polyphenols remains limited because of their reduced activity due to their interactions with food components, low stability, poor standardization, undesirable sensorial properties, and regulatory issues. Considering the recent studies, some approaches such as micro- or nanoencapsulation, active packaging, and edible coating and films can overcome the aforementioned limitations. However, very few studies assessed the effect of polyphenols on different type of food products using these approaches. Hence, further investigations are required to compare the efficiency of these techniques, and to determine the best approaches to deliver antimicrobial polyphenols with maximum potential. It is noteworthy that most of the polyphenols, particularly at high dose, exhibit toxic effects and strictly warrant the determination of safety concerns upon ingestion of food materials with such antimicrobials.

## Figures and Tables

**Table 1:** A representation of antimicrobial polyphenols from flavonoid and non-flavonoid classes with food preservative potential.

Flavonoid Class of Antimicrobial Polyphenols				
Compounds	Plant source	Bioactivity	Pathogens (MIC ^1^ Value in µg mL^−1^)	References
Flavones				
Amentoflavone	*Dorstenia barteri*	Antimicrobial	*B. cereus*, *B. subtilis, S. dysenteriae, C. albicans, C. glabrata* (>39.1)	[20]
Baicalein	*Scutellaria baicalensis*	Anti-quorum sensing, Antibiofilm (modulating the expression of *sarA* and *ica* operon)	*S. aureus* (32 and 64)	[21]
Chrysin derivative 8c	*Oroxylum indicum*	Antibacterial, targeting β-ketoacyl-acyl carrier protein synthase III (FabH protein)	*E. coli* (6.25)	[22,23]
Diosmetin	*Sophora moorcroftiana*	Anti-virulence	*S. aureus* (32 and 64)	[24]
		Antibacterial, modulate the expression of ATP ^2^ Binding Cassette (ABC ^6^) transporter via synergistic action with erythromycin	*S. aureus* RN4220 (diosmetin (8) and erythromycin (32) with FICI ^3^ value of 0.28)	[25]
Gancaonin Q	*Dorstenia angusticornis*	Antimicrobial	*B. cereus* (2.4), *B. subtilis* (9.76), *Shigella dysenteriae* (2.44), *Shigella flexneri* (19.53), *Salmonella* Typhi (39.06), and for *C. albicans Candida krusei* and *Candida glabrata* (>78.12)	[26]
Licoflavone C	*Retama raetam*	Antibacterial	*E. coli* (7.8)	[27]
Luteolin	*Elsholtzia rugulosa*	Antibacterial, via inhibiting DNA topoisomerase	*S. aureus* (1.6 mg mL^−1^)	[28]
6-Prenylapigenin	*Dorstenia* sp.,	Antimicrobial, rapid killing activity via depolarizing the cell membrane and inhibiting the biosynthesis of DNA, RNA and proteins	*S. aureus* (16 and 32) MRSA ^4^ (16–64) *C. albicans* (64)	[29]
**Isoflavones**				
Biochanin A	*Lycium barbarum*	Antibacterial (Strain dependent activity)	*Clostridium perfringens* (64–1024)	[30]
Daidzein	*Glycine max*	Antibacterial	*Listeria monocytogenes* and *Vibrio parahaemolyticus* (125 µM ml^−1^), *B. cereus, S. aureus* and *S*. Typhimurium (500 µM ml^−1^)	[31]
Genistein	*Glycine max*	Antibacterial, involve the stabilization of the covalent topoisomerase II-DNA cleavage	*L. monocytogenes* and *V. parahaemolyticus* (125 µM ml^−1^), *Helicobacter pylori*, *S. aureus, B. cereus* (100 µM ml^−1^)	[31,32]
Isolupalbigenin	*Erythrina poeppigiana*	Antibacterial	MRSA ^4^ (1.56–3.13)	[33]
**Flavonols**				
Galangin	*Helichrysum aureonitens*	Antibacterial	*S. aureus* (50)	[34]
Kaempferol	*Glycine max*	Antibiofilm, altering the activity of sortase enzyme and adhesin related gene expression	*S. aureus* (64)	[35]
Morin	*Psidium guajava*	Antibiofilm and anti-virulence	*L. monocytogenes* (25) *C. albicans* (150)	[36,37]
		Antibacterial	*L. monocytogenes* (100)	
Myricetin	*Myrica rubra*	Antibiofilm	MRSA ^4^ (32) VISA ^5^ (16) *S. aureus* (32)	[38]
Quercetin	*Olea europaea*	Antibacterial	*E. coli*, (0.0082 µM mL^−1^) *S.* Typhimurium (0.0072 µM mL^−1^) and *S. aureus* (0.0068 µM mL^−1^)	[39]
Quercetin-3-O-rhamnoside	*sCapsicum annuum*	Antibiofilm	S. Typhimurium, and *S. aureus* (1 mg ml^−1^)	[40]
		Antibacterial	S. Typhimurium, S. Enteritidis, *E. coli*, *S. aureus*, *C. jejuni*, *Stenotrophomonas maltophilia*, *Klebsiella pneumoniae* and *Enterobacter cloacae* (0.03 to 1.25 mg ml^−1^)	
Rutin	*Olea europaea*	Antibacterial, both at mono and multispecies level	*E. coli* and *S. aureus* (400–1200)	[41]
**Flavanones**				
Diplacone	*Paulownia tomentosa*	Antibacterial	MRSA^4^ (2–16)	[42]
Mimulone			MRSA^4^ (8–64)	
6, 8- Diprenyleriodictyol	*Dorstenia* sp.,	Antimicrobial, rapid killing via depolarizing the cell membrane and inhibiting the biosynthesis of DNA, RNA and proteins	*S. aureus* (0.5 and 4) MRSA ^4^ (1–4) *C. albicans* (128)	[29]
Naringenin	*Citrus paradisi*	Antibiofilm	*S.* Typhimurium (30) *Lactobacillus rhamnosus* (30) MRSA (200)	[43,44]
Pinocembrin	*Glycyrrhiza glabra*	Anti-virulence, reducing α-toxin mediated cell injury in mouse model by reducing α-toxin production	*S. aureus* (64 to >128)	[45]
Sakuranetin	*Baccharis retusa*	Antifungal	*Candida dubliniensis, Candida tropicalis, C. glabrata, Candida parapsilosis* and *C. krusei* (0.63) *C. albicans* and *Cryptococcus gattii* (0.32) *C. neoformans* (0.08 and 0.32) *Saccharomyces cerevisiae* (0.32)	[46]
**Flavanols**				
Catechins	*Camellia sinensis*	Antibacterial	*B. subtilis* and *E. coli* (9 ppm)	[47]
Epicatechin	*Malus domestica*	Antibacterial	*B. subtilis, B. subtilis, C. perfringens* (20 ppm) *L. monocytogenes* (5–20 ppm)	[48]
Epicatechin gallate	*Camellia sinensis*	Anti-virulence, targeting α-toxin, coagulase and protease activities	*S. aureus* (25)	[49]
Epigallocatechin gallate	*Camellia sinensis*	Antibacterial, hinder the functions of membrane proteins, such as oligopeptide ABC^6^ transporter, phosphotransferase system transporter, phosphate ABC^6^ transporter, and penicillin binding protein 5	*B. subtilis* JCM1465 ^7^ (125) *B. subtilis* 168 (250)	[50]
**Chalcones**				
4-Hydroxyonchocarpin	*Dorstenia* sp.,	Antibacterial	*S. aureus* (1–8)	[29]
licochalcone A	*Glycyrrhiza* sp.,	Antibiofilm and inhibit the yeast-hyphal transition	*C. albicans* (0.2)	[51]
licochalcone E	*Glycyrrhiza inflata*	Antibacterial	*S. aureus* (1-4)	[52]
Isobavachalcone	*Psoralea corylifolia*	Antibacterial	*S. aureus* (0.3)	[29]
**Non-Flavonoids classes of polyphenols**				
**Phenolic acids**				
Chlorogenic acid	*Coffea* sp.,	Anti-virulence, targeting sortase enzyme	*S. aureus* (33.86 ± 5.55)	[53]
		Antibacterial, leading cell death by targeting ROS mediated cell signaling	*E. coli* (64)	[54]
ρ-Coumaric acid	*Vitis vinifera*	Antibacterial	*Lactobacillus hilgardii* (500)	[55]
Ferulic acid	*Beta vulgaris*	Antibacterial	*Cronobacter sakazakii* (2.5–5.0 mg mL^−1^) *E. coli* (1.5 mg mL^−1^) *S. aureus* (1.75 mg mL^−1^) and *L. monocytogenes* (2.0 mg mL^−1^)	[56,57]
Gallic acid	*Vaccinium corymbosum*	Antibacterial	*E. coli* (0.1 mg mL^−1^) *S. aureus* (1.25 mg mL^−1^) and *L. monocytogenes* (1.25 mg mL^−1^)	[56]
ρ-Hydroxybenzoic acid	*Macrotyloma uniflorum*, *Cocos nucifera*	Antibacterial, displayed pH dependent activity in *L. monocytogenes* and exposure of higher MIC^1^ not develop resistance to antibiotics in *S. aureus*	*L. monocytogenes* (5 and 10 mM mL^−1^) *S. aureus* (1.6 mg mL^−1^)	[58,59]
Vanillic acid	*Angelica sinensis*	Antibacterial, Exposure of higher MIC not develop resistance to antibiotics	*S. aureus* (2.5 mg mL^−1^)	[59]
**Stilbenes**				
Combretastatin B5	*Combretum woodii*	Antibacterial	*S. aureus* (16 mg mL^−1^)	[60]
Longistylin A	*Cajanus cajan*	Antibacterial, rapid activity by disturbing membrane potential and improved healing in infected mice wound model	MRSA ^4^ (1.56)	[61]
Resveratrol	*Vitis vinifera*	Antibacterial	MRSA ^4^ (32–260) *S. aureus* (350) *B. cereus* ATCC11778 ^8^ (52) and TISTR687 ^9^ (64) *L. monocytogenes* and *L. innocua* (200) *E. coli* (32–521) *S.* Typhimurium (5–500) *V. cholera* ATCC39315 ^8^ (0.625) and MCVO9 (60) *C. coli* (50) *C. jejuni* (100–313) *Arcobacter butzleri* (100) *Arcobacter cryaerophilus* (500)	[62]
**Lignan**				
Sesamin	*Sesamum indicum*	Antibacterial	*S. aureus* and *B. cereus* (2 mg mL^−1^)	[63]
Justicidin B	*Phyllanthus piscatorum*	Antifungal	*Aspergillus fumigatus* (1) *Aspergillus flavus* (12) and *C. albicans* (4)	[64]
Sesamol	*Sesamum indicum*	Antifungal	*Mucor circinelloides,Aspergillus niger, S. cerevisiae, Aspergillus flavipes, Candida utilis,* and *Cryptococcus curvatus* (7.2 mM mL^−1^)	[65]
Sesamolin	*Sesamum indicum*	Antibacterial	*S. aureus* and *B. cereus* (2 mg mL^−1^)	[63]
**Coumarins**				
Umbelliferone	*Rhododendron lepidotum*	Antibacterial	*S. aureus* (500) MRSA^4^ and E. coli (1000)	[66]
Xanthotoxin	*Heracleum mantegazzianum*	Antimicrobial	*S. aureus* (0.03–0.25) *B. subtilis* (0.03) *B. cereus* (0.5) *Micrococcus luteus* (0.03) *E. coli* (1.0) *S.* Typhimurium (1.0) *C. albicans* (0.125–0.25) *Candida parapsilosis* (0.06)	[67]
**Tannins**				
Casuarictin	*Casuarina stricta*	Antibacterial	*H. pylori* (12.5)	[68]
Geraniin	*Geranium thunbergii*	Antibacterial	*H. pylori* (12.5)	
Oenothein A	*Oenothera stricta*	Antibacterial	*H. pylori* (50)	
Oenothein B	*Oenothera stricta*	Antibacterial	*H. pylori* (25)	
Pedunculagin	*Agrimonia pilosa*	Antibacterial	*H. pylori* (12.5)	
Penta-O-galloyl-β-D-glucose	*Eucalyptus* sp.,	Antibacterial	*H. pylori* (12.5)	
Procyanidin B-1	*Vitis vinifera*	Antibacterial	*H. pylori* (100)	
Procyanidin B-3	*Vitis vinifera*	Antibacterial	*H. pylori* (100)	
Tannic acid	*Quercus* sp.,	Anti-virulence, manipulate the expression of hemolysin production and biofilm formation	*S. aureus* (50)	[69]
		Antibacterial	MRSA^4^ and *S. aureus* (40–160)	[70]
Punicalagin	*Punica granatum*	Antibacterial	*S. aureus* (163–200) *Salmonella arizonae* (800) *Salmonella anatum* (800) *Salmonella* serotype O (600–800) *E. coli* (IFO-3.2; ATCC25922 ^8^—1.6 mg mL^−1^) Nonpathogenic *E. coli* (2.1 mg mL^−1^) Enterohemorrhagic *E. coli* (1.44 mg mL^−1^) Enteroinvasive *E. coli* (1.0 mg mL^−1^) Enterotoxigenic *E. coli* (1.6 mg mL^−1^) *V. cholera* (100) *V. parahaemolyticus* (60–100) *Vibrio vulnificus* (45)	[71]
**Quinones**				
Cryptotanshinone	*Salvia miltiorrhiza*	Antibacterial	MRSA ^4^ (4–64) MSSA ^10^ (16–64) VRSA ^11^ (2–4)	[72]
6-(4, 7 dihydroxy-heptyl) Quinone	*Pergularia daemia*	Antibacterial	*S. aureus* (75), *B. subtilis* (50)	[73]
**Curcuminoids**				
Curcumin	*Curcuma longa*	Antibacterial	MRSA ^4^ (129) MSSA ^10^ (219) *B. subtilis* (217)	[74]
Curcumin-β-diglucoside	Derivative of curcumin	Antibacterial	*B. cereus* (0.181 µM), *S. aureus* (0.051 µM), *E. coli* (0.469 µM) and *Y. enterocolitica* (0.867 µM)	[75]
Demethoxycurcumin	Derivative of curcumin	Antibacterial	*E. coli* (512), *S. dysenteriae* (1024) and *S. aureus* (1024)	[76]
**Xanthanoids**				
α-Mangostin	*Garcinia mangostana*	Antibacterial	MRSA ^4^ (0.78) MSSA ^10^ (1.56)	[77]

Abbreviations: ^1^ MIC: minimum inhibitory concentration; ^2^ ATP: adenosine triphosphate; ^3^ FICI: fractional inhibitory concentration index; ^4^ MRSA: methicillin-resistant *S. aureus*; ^5^ VISA: vancomycin intermediate resistant *S. aureus*; ^6^ ABC: ATP-binding cassette; ^7^ JCM: Japan Collection of Microorganisms; ^8^ ATCC: American Type Culture Collection; ^9^ TISTR: Thailand Institute of Scientific and Technological Research; ^10^ MSSA: methicillin sensitive *S. aureus*; ^11^ VRSA: vancomycin resistant S. aureus.

**Table 2:** Recent studies showing the application of polyphenols rich PE or alone antimicrobial polyphenols in food packaging.

Antimicrobial PE/Polyphenols	Method	Carrier	Results	References
Carvacrol	Encapsulation (electrospinning)	Poly-ε-caprolactone fibers	The resulting fiber (ca.200 nm in length) accommodate 11 g of drug per 100 g of fiber with encapsulation efficiency of 85%. The resulting fibre showed a controlled release drug over the period of test time. The fiber showed better activity against *E. coli* than *L. innocua.* Antimicrobial effect of the fiber not only depend on the drug alone but also its release capacity.	[135]
Curry plant EO	Encapsulation (thin-film dispersion)	Soy lecithin/cholesterolliposomes	The average size of liposomal nanocarrier was 196 nm. The entrapment efficiency of the liposome nanocarrier was 56.34% One mililitre of nanocarrier was reported to accommodate 2–6 mg of the drug Drug loaded liposomal nanocarrier at 20% (*v v*^−1^) significantly reduced the *B. cereus* burden in the rice flour food model.	[136]
Eugenol	Encapsulation (ultrasonication-mediated emulsification)	Chitosan	The size of the chitosan nanoparticles ranges between 215.5–794.4 nm. The encapsulation efficacy of chitosan nanoparticle was 11.61%. The MIC value of eugenol was reduced when used with chitosan nanoparticles, suggesting the potential of encapsulation in improving the bioactivity of the drug against *S. aureus*, *E. coli* O157:H7, *P. aeruginosa*, *Salmonella*. The prepared encapsulaed drug also showed potent antioxidant activity.	[137]
Sour cherry oil	Encapsulation (spray-drying)	Maltodextrin/ gum Arabic	The average paticle size of the encapsulated sour cherry oil is 10 μm. The encapsulation efficacy of the nanoparticle was approximately 89%. In thermal stability assay, the prepared nanoparticles surpasses 200 °C without any pronounced loss in mass. The resulted nanoparticles showed profound growth inhibitory activity against the tested MOs such as *S. aureus*, *P. aeruginosa*, *E. faecalis*, *C. albicans.*	[138]
Epigallocatechin gallate (EGCG ^1^)	Edible coating (solution-casting method)	Sodium alginate (SA) and carboxymethyl cellulose (CMC)	EGCG ^1^ at 1.6% (*w v*^−1^) in SA-CMC solution prevent the pork samples from weight loss. The EGCG ^1^ edible coating prevent the pork from early decay by inhibiting the total viable counts. The resultant coating enhanced the shelf-life of the fresh pork. The sensory attributes of the pork coated with EGCG ^1^ were significantly improved. Moreover, the drug coatings significantly reduced the lipid peroxidation and total volatile basic nitrogen.	[139]
Catechin/ nisin	Edible coating (solution-casting method)	Gelatin	The gelatin films incorporating nisin (0.12% *w w*^−1^)/ nisin-catechin combinations (0.06% *w w*^−1^, each) were effective against *S. aureus* and *B. cereus.* In the 7-days experimental period, the gelatin films were improved the quality attributes of minced pork. During this period, it was also found that the drug loaded gelatin films reduced the value of thiobarbituric acid reactive substances in the minced pork.	[140]
Oregano EO (OEO ^2^)/ resveratrol (RES ^3^)	Edible coating (nanoemulsion)	Pectin	The initial particle size of the OEO ^2^ and OEO ^2^-RES ^3^ nanoemulsions, and OEO ^2^-RES ^3^ emulsions were found to be 48.49, 53.09 and 220.01 nm, respectively. OEO ^2^ and RES ^3^ nanoemulsions were showed a better stability at 4 °C for 15 days The pork loins coated with OEO ^2^ and RES ^3^ nanoemulsion showed an increased shelf-life by reducing the pH and colour change, delaying the oxidation of lipid and protein, maintaining meat tenderness, and inhibiting microbial growth, i.e., total viable counts.	[141]
OEO ^2^/Bergamot PEO ^4^	Edible coating	Hydroxypropyl methylcellulose	Coating containing 2% (*v v*^−1^) OEO ^2^ showed good antibacterial activity against *E. coli.* Coating containing 2% (*v v*^−1^) OEO^2^ extended the freshness of the food model plum, Prunus salicina. The coating containing the PEO^4^ did not affect the organoleptic properties of the plum.	[142]
Rosemary PEO	Edible coating	Whey protein (Glycerol as plasticizer)	One percent (*w w*^−1^) of rosemary PEO^4^ inhibit the growth of the test pathogens, *L. monocytogenes* and *S. aureus.* The coating also showed good antioxidant activity.	[143]
Thymol and Carvacrol	Active packaging	Low density polyethylene (LDPE ^5^) with hallosyte nanotubes	The LDPE^5^ films showed potent and prolonged antimicrobial activity in lab conditions and in real food system hummus spread. Comparing the individual durg stability and growth inhibitory activity against *E. coli* exerted by T ^6^/C ^7^ mixture was higher. Compared with the activity of reference film, the T ^6^-C ^7^ containing films have completely eradicated the *E. coli* growth in hummus spread. The films were able to retain their activity for the time of 8 weeks.	[144]
Cranberry extract	Active packaging	Chitosan	Food preservative properties such as light penetration, and permeability to water and oxygen were apparent in film incorporating cranberry extract. The cranberry extract incorporated films exerts antimicrobial and antibiofilm activities against the tested MOs such as *S. aureus* and *E. coli.*	[145]

Abbreviations: ^1^ EGCG: Epigallocatechin gallate; ^2^ OEO: oregano essential oil; ^3^ RES: resveratrol; ^4^ PEO: plant essential oil; ^5^ LDPE: low density polyethylene; ^6^ T: thymol; ^7^ C: carvacrol.

## Data Availability

Not applicable.
